# Creative music therapy to promote brain structure, function, and neurobehavioral outcomes in preterm infants: a randomized controlled pilot trial protocol

**DOI:** 10.1186/s40814-017-0180-5

**Published:** 2017-09-26

**Authors:** Friederike Barbara Haslbeck, Hans-Ulrich Bucher, Dirk Bassler, Cornelia Hagmann

**Affiliations:** 1 Department of Neonatology, University Hospital Zurich, Frauenklinikstrasse 10, 8091 Zurich, Switzerland; 2 Department of Pediatric Intensive Care and Neonatology, University Children’s Hospital, 8032 Zurich, Switzerland

**Keywords:** Preterm infants, Brain development, Creative music therapy, Randomized controlled trial, Neurobehavioral outcomes

## Abstract

**Background:**

Preterm birth is associated with increased risk of neurological impairment and deficits in cognition, motor function, and behavioral problems. Limited studies indicate that multi-sensory experiences support brain development in preterm infants. Music appears to promote neurobiological processes and neuronal learning in the human brain. Creative music therapy (CMT) is an individualized, interactive therapeutic approach based on the theory and methods of Nordoff and Robbins. CMT may promote brain development in preterm infants via concurrent interaction and meaningful auditory stimulation. We hypothesize that preterm infants who receive creative music therapy during neonatal intensive care admission will have developmental benefits short- and long-term brain function.

**Methods/design:**

A prospective, randomized controlled single-center pilot trial involving 60 clinically stable preterm infants under 32 weeks of gestational age is conducted in preparation for a multi-center trial. Thirty infants each are randomized to either standard neonatal intensive care or standard care with CMT. Music therapy intervention is approximately 20 min in duration three times per week. A trained music therapist sings for the infants in lullaby style, individually entrained and adjusted to the infant’s rhythm and affect. Primary objectives of this study are feasibility of protocol implementation and investigating the potential mechanism of efficacy for this new intervention. To examine the effect of this new intervention, non-invasive, quantitative magnetic resonance imaging (MRI) methods at corrected age and standardized neurodevelopmental assessments using the Bayley Scales of Infant and Toddler Development third edition at a corrected age of 24 months and Kaufman Assessment Battery for Children at 5 years will be performed. All assessments will be performed and analyzed by blinded experts.

**Discussion:**

To our knowledge, this is the first randomized controlled clinical trial to systematically examine possible effects of creative music therapy on short- and long-term brain development in preterm infants. This project lies at the interface of music therapy, neuroscience, and medical imaging. New insights into the potential role and impact of music on brain function and development may be elucidated. If such a low-cost, low-risk intervention is demonstrated in a future multi-center trial to be effective in supporting brain development in preterm neonates, findings could have broad clinical implications for this vulnerable patient population.

**Trial registration:**

ClinicalTrials.gov, NCT02434224.

**Electronic supplementary material:**

The online version of this article (10.1186/s40814-017-0180-5) contains supplementary material, which is available to authorized users.

## Background

Infants born prematurely (i.e., < 37 weeks gestation) are a growing patient population representing an important sector of pediatric health care. Globally, 15 million infants a year are born prematurely [1]. Indeed, approximately 1 in 10 babies is born prematurely in the USA and Europe [2]. Notably, preterm birth is a major determinant of both neonatal mortality and morbidity that can have long-term adverse health consequences. Premature infants are particularly vulnerable to brain injury, and preterm birth is associated with reduced white and gray matter volumes. With regard to gray matter, particular regions appear to be affected. Decreased thalamic volume [3], cortical gray matter volume [4], and surface gyrification [5] have been observed. These abnormalities are thought to result from periventricular white matter injury (PWMI)—the predominant form of perinatal brain injury among preterm infants [6]. Such brain structure abnormalities persist into later life and have been linked to a range of neurodevelopmental impairments including cerebral palsy, motor dysfunction, and cognitive and behavioral problems as well as deficits in executive function [7].

Strikingly, those children/adolescents who were born preterm, yet who have normal intellectual and motor function, are at increased risk for deficits in executive function such as attentional control, working memory, reasoning, and planning [8]. Factors such as environmental noise and medically required isolation may induce high levels of stress in preterm infants which may compound the impaired neurodevelopment [9–11]. It has been posited that brain maturation is effected by multiple factors. For instance, the sometimes overwhelming auditory environment of the neonatal intensive care unit (NICU) may be problematic while conversely, auditory deprivation (e.g., the lack of the regular intrauterine rhythms of the maternal heart beat and the maternal voice) may also impact maturation [12–14].

### Neurobiology of music during early life

A growing area of interest is how interactive, multi-sensory experiences during fetal life may improve brain development in the fetus [15–17]. Both human and animal studies demonstrate that early auditory experiences can influence brain development [18–20]. This is particularly important following preterm birth as the plasticity of auditory regions, and cortex development is heavily dependent on the quality of auditory experiences [21]. Angelucci and colleagues [22] showed that exposing young adult mice to music can stimulate neuron development and increased levels of nerve growth factors in the hippocampus, hypothalamus, and cortical areas. Thus, it is speculated that positive auditory experience may promote the preterm infants’ early brain maturation and contribute to subsequent neurodevelopment [13, 23, 24]. Studies at the interface of music science and neuroscience have demonstrated that music promotes neurobiological processes in humans including modulation of synaptic plasticity, neuronal learning, and neuronal readjustment [25–27]. For instance, music can change the brain activity in core structures involved in processing emotions [28]. Therefore, it appears that the individualized approach of live music therapy may activate various brain regions involved in emotional, sensorimotor, and cognitive processing, e.g., perception-action mediation in premotor areas, emotional modulation within the limbic system, and intentional processes of social cognition in frontal and temporal regions [29–31].

Auditory input appears to be critical for brain maturation development in early life beginning in utero, into the newborn period, and through early neonatal life. Prior studies have shown that musical learning begins prior to birth [32–35] and extensive prenatal exposure to a melody induces neural representations that can last several months [36]. In neonates, music stimulation triggers neuronal activation in the bilateral front lobes [32]. Moreover, neuroimaging studies have identified that music activates limbic and paralimbic regions in the human brain and this engagement may enhance psychological and physiological health [37, 38].

### Potential benefits of creative music therapy

Creative music therapy (CMT) is an interactive needs-oriented approach based upon the theory and methods of Nordoff and Robbins [39]. This framework has been adapted to address the specific needs of preterm infants and their parents in the NICU setting [40, 41]. Using this approach, the music therapist interacts with the infant through improvised, entrained humming/singing. The music therapist assesses the infant’s respiration, as breathing is the most fundamental observable human rhythm, in concert with the infant’s facial expressions and gesticulations. The therapist’s response is an infant-directed, improvised humming that is continually adapted and tailored to the infant’s needs based on the dynamic rhythms and subtle expressions of the infant. This often occurs in a synchronous manner, e.g., when the infant’s eyebrows lift, the music therapist responds by steering the melody pitch and tempo upward [41]. Conversely, when the infant is overly aroused, the therapist may shift the melody downwards, slowing the tempo to soothe the baby with calming musical tones [42]. The aim is to keep the infant-directed humming/singing as simple as possible, as preterm infants can easily become overstimulated by sensory stimuli.

The humming is characterized by smooth, fluent melody contours using rich overtones as in a lullaby and maintaining an infant-directed style. The focus is to keep the music calm, simple, predictable, and repetitive—in line with existing recommendations and guidelines on music therapy in the NICU setting [43]. Whenever possible, willing parents are engaged and involved in the therapeutic process, e.g., by providing music therapy during kangaroo care (i.e., skin-to-skin holding) and by supporting and encouraging them to sing to their infant in this responsive and interactive manner to foster an intuitive parent-infant interaction contributing to the bonding process. CMT incorporates psychological models of traumatization and includes targeted interventions to facilitate relaxation, coping and developing a healthy parent-infant relationship [44].

CMT can be used to calm preterm infants and for respiratory stabilization. Positive music therapy outcomes (i.e., arousal, behavior, respiratory rate) have been shown in a multi-site trial as well as in a systematic review and meta-analysis [45–47]. Our previous work has demonstrated that the responsive experiences with CMT help guide infants in engaging in meaningful, nurturing interactions [48].To date, a significant gap in our understanding of CMT has been the paucity of data examining the effects over time. The recent meta-analysis highlighted the need for long-term investigation of CMT interventions for neonates implemented by specially trained music therapists [45]. Indeed, most studies have only focused on short-term effects, were not randomized or controlled, and included relatively small sample sizes. Thus, more rigorous, well-designed studies (i.e., randomized controlled trials) are warranted to further elucidate the respective benefits and limitations of CMT in neonatal care.

**Fig. 1:**
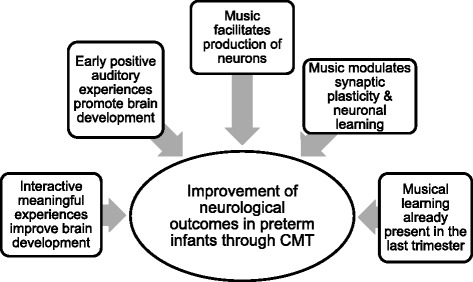
Factors leading to the development of hypotheses

A critical question is whether or not the individualized socio-emotional and auditory interactive experiences of creative music therapy can be quantified in promoting brain development in preterm infants (Fig. 1). Interestingly, regions for music and language are strongly interconnected in the brain [30, 49]. While the mechanism for such a process is not yet fully elucidated, it is tempting to speculate that the enriched experience and environment created by individualized CMT will promote brain development in preterm infants and that these critical early developmental events will have developmental benefits in short- and long-term brain function (i.e., language acquisition).

To address this gap in the field, we conduct a randomized controlled pilot trial examining the short- and long-term neurodevelopmental and neurobehavioral outcomes of therapeutic CMT treatment. We hypothesize that compared to controls, preterm neonates receiving the CMT intervention will demonstrate:

- Improved brain growth and development at 38–42 weeks (corrected gestational age) as evidenced by
  - Greater global and regional brain volumes measured by 3D volumetric magnet resonance imaging (MRI)
  - Enhanced white matter microstructure (higher fractional anisotropy)
  - Improved functional network development
- Improved cognitive, behavioral and motor developmental outcome at 24 months and 5 years in terms of adaptive skills, executive function, and language abilities using validated metrics.

## Methods

### Design and setting

The pilot study is designed as a prospective, single-center, randomized, controlled clinical trial in preparation for a full-scale trial. Since this is the first study to evaluate possible effects of CMT on neurobehavioral outcomes in preterm infants, a pilot design is chosen to test its clinical, recruitment, and outcome measurement feasibility [50]. In total, 60 preterm infants will be included in the study since a minimum of 30 participants for each arm in pilot studies is recommended in order to estimate parameters for future sample size calculations [50, 51].

The inclusion criteria are as follows: gestational age at birth < 32 weeks (due to a necessary time frame of 4 weeks at a minimum for music therapy treatment), chronological age ≥ 7 days of life, and clinically stable and signed, written parental informed consent. Neonates with a genetically defined syndrome, congenital malformation (adversely affecting life expectancy or neurodevelopment), moderate/severe intraventricular hemorrhage, congenital hearing loss, or those infants admitted for palliative care are excluded from the study. Preterm neonates are excluded from the control group in situations when infants are exposed to regular singing (or other kinds of music stimulation) from parents during hospitalization.

All parents of neonates meeting the inclusion criteria are asked by the investigator to participate in the study within the second weeks after birth, i.e., day of life 7–14 (Additional file 1). Following signed informed consent (Additional file 2), randomization is performed using a computer-generated list created prior to study initiation (Table 1). Neonates randomized to the control group receive standard care as delivered by the Department of Neonatology at the University Hospital of Zurich. Of note, and as stated in the exclusion criteria, standard neonatal care does not include any type of music stimulation for the preterm infant.

**Table 1:** Course and outcome measures of music trial

Screening	Enrollment and allocation		Outcome measures		
Birth until DOL 7	DOL 7–14	DOL 8 until discharge	At TEA CGA	24 months CGA	5 years
Inclusion and exclusion criteria	Informed consent Randomization	Intervention group: CMT 3×/week and standard care control group: standard care	Brain MRI	BSID-III	Kaufman ABC
	cUS	cUS weekly		Neurological examination	Neurological examination
	Clinical records for morbidities, SES	Clinical records for morbidities, SES		Parental questionnaire	Parental questionnaire

### The intervention

Experimental group infants receive the CMT intervention. The individualized therapeutic approach is delivered as initially reported [48] and as described above. CMT therapy is initiated immediately following the parental consent. Therapy sessions are delivered three times per week mostly in the morning until discharge by a music therapist (FH). To ensure the safety of the infants, the chosen study music therapist is a well-trained and experienced music therapist with specialized competencies, sensitivity, and responsiveness in neonatal music therapy [52]. CMT with premature infants is based upon the premise that infants should be neither overwhelmed nor over-stimulated [41]. However, in the case the infants would show adverse reactions (e.g., unexpected strong apnea, bradycardia, hypoxia) during the music therapy intervention, the music therapist would adapt or stop the treatment immediately. Moreover, the nurse in charge continuously monitors the therapy process to additionally guarantee the safety of the participants.

Individualized, culturally adapted treatment plans are formulated based on an initial child-parent assessment including consideration of stated parental needs, musical heritage, culture, context, and desire for integration into the therapeutic process. Emphasis is placed on providing a supporting role rather than assuming a directive, educational, or corrective role with respect to parental competencies and autonomy. In general, each CMT intervention lasts approximately 20 min. Duration is adapted according to infant needs and development—both immediate and evolving. We expect sessions increase in length as the infant matures. The exact number, time, and duration of sessions are documented in the individual session protocol and final report (Additional file 3). To maintain study enrollment, neonates randomized to receive the CMT intervention must receive a minimum of ten sessions of CMT—which is the number of sessions needed to measure an effect of the therapy [42, 43]. Prior to hospital discharge, the music therapist provides a final consultation and debriefing discussion with the parents including therapeutic recommendation and offering music therapy consultation for the first year of life.

Preterm infants have to be excluded from the study if they are not stable enough for CMT treatment and/or would show serious behavioral or physiological signs of overstimulation or other adverse reactions during or after the music therapy intervention. Moreover, the sponsor continuously monitors the therapy process to guarantee the safety of the participants. The sponsor can terminate the study within its area of responsibility in the case of threatening the patient’s well-being and safety. Under such circumstances, the ethics committee will be informed immediately.

### Minimizing bias

The music therapy process is documented by the music therapist in a standardized form (Additional file 3) in detailed reflections depicting methodical and therapeutic procedures as well as notes recording parental involvement, environmental circumstances, and additional objective physiologic measures (respiration and heart rate, oxygen saturation) recorded by ongoing electronic monitoring. An independent study nurse randomly monitors this process to minimize bias. To maintain blinding for the subsequent long-term follow-up assessment (at 2 and 5 years) of primary and secondary neurodevelopmental outcomes, no notes of the CMT are entered into the medical record.

Central randomization was performed by the Clinical Trial Center Zurich on the basis of randomization lists. Selection bias is minimized with allocation concealment and random sequence. Sealed, opaque, numbered envelopes are opened sequentially only after the envelope has been irreversibly assigned to the participant. The allocation process is monitored by the study sponsor to preserve concealment. In the event that standard care for an infant in the control group could be contaminated (i.e., intervention and control have adjacent cribs), the neonate in the control group receives hearing protection via ear plugs and special headsets for premature infants placed during the music therapy session. The hearing protection device employed for this purpose effectively blocks 20–30 dB of environmental sound. This corresponds with the highest possible decibel level produced at the bedside during the music therapy intervention (measured with “dB Volume, DSP Mobile, Iphone App”). Prior to discharge, parents of infants in the control group are interviewed if infants were exposed to singing or another kind of music stimulation during hospitalization. Only the control infants who have received no humming/singing/musical stimulation are included in the analysis as the comparison group. The study protocol received approval by the independent SwissPharmAudit (audit no.: CTCQA14).

### Outcomes

The primary objective of this study is feasibility of protocol implementation, including the estimation of drop-out rate and differential drop-out rate within treatment groups, and further objectives of the study are investigating the potential mechanisms of efficacy for this new intervention. The potential mechanisms of efficacy are measured with cerebral magnetic resonance imaging and neurodevelopmental outcome at corrected 2 and 5 years of age.

Advantages of using MRI are that it is non-invasive, quantitative, sensitive measurement. Microstructure of the white matter is assessed using tract-based spatial statistics (TBSS), an automated, observer-independent method for assessing fractional anisotropy in the major white matter tracts on a voxel-wise basis across groups of subjects [9]. TBSS has been used to identify subtle group differences in studies of patients with birth weight [53] or neuroprotective interventions [54]. Brain morphology is assessed both globally (intracranial cavity, cerebellum, brainstem, two hemispheres) and at the tissue level (cortical and subcortical gray matter, myelinated and unmyelinated white matter, cerebrospinal fluid) segmentation [55] to evaluate group differences. Functional and structural connectivity is compared between the groups using diffusion tensor imaging (DTI) and resting state functional magnetic resonance imaging (rsFMRI).

Neurodevelopmental and neurological outcome will be assessed with (Table 1):

- Bayley Scales of Infant and Toddler Development III (BSID-III) including mental development index and psychomotor development index assessment [56]
- Neurological exam (including classification of cerebral palsy in Europe) and Palisano’s gross motor function classification [57, 58]
- Visual and hearing exam [59]
- Adaptive Behavior Assessment System (ABAS)-II parental questionnaire which is a comprehensive assessment of daily adaptive skills necessary for functioning effectively and navigating one’s environment based on typical demands according to age [60]

Additionally, a parental questionnaire assessing the infant’s exposure to music is administered.

Since there is a substantial gap in present knowledge to assess the long-term impact of music therapy in preterm infants [45], and since at 2 years of age merely developmental milestones can be measured while more complex cognitive functions such as executive functions are only developing, we assess neurodevelopmental outcomes also at 5 years of corrected age [61]. Along these lines, it was recently postulated that the 2-year outcome should be used as a proof for safety of neuroprotective agents rather than their efficacy [61]. Hence, following tests will be performed at 5 years of corrected age relating to six main neurobehavioral outcomes including (Table 1):

- Kaufman Assessment Battery for Children (KABC)-II [62] to evaluate cognitive development
- Neurological exam with cerebral palsy qualification and Palisano’s gross motor function classification [57]
- Behavior exam: Strengths and Difficulties Questionnaire (SDQ) evaluating physical limitations [63]
- Gross motor exam “Zürcher Neuromotorik” to assess balance, gaits, speed, and mirror overflow (referred to as “associated movements,” AM) [57]
- Assessments of visual problems such as corrective glasses, strabism, severe visual impairment, or blindness [64]
- Assessment of hearing problems such as moderate hearing impairment not requiring hearing aids or cochlear implant [64]
- Parental questionnaires:
  - Adaptive Behavior Assessment System (ABAS)-II for assessing of daily adaptive skills [60]
  - Behavior Rating Inventory of Executive Function - Preschool Version (BRIEF-P) to report on executive function abilities [65]

Additionally, a parental questionnaire about their (musical) education of their infant will be distributed.

All listed neurobehavioral outcomes from 18 months to 5 years except the parental questionnaires ABAS-II and BRIEF-P are validated routine assessments within the Swiss Neonatal Network. These assessment tools have been standardized for follow-up assessments and are used by child development specialists in Switzerland since 2006 and thus reflect Swiss consensus on optimal follow-up assessment for premature infants [64]. These instruments are accepted and widely used within the Swiss Neonatal Network (http://www.neonet.ch/en/). To ensure high follow-up rates, all parents of premature infants are invited to the compulsory follow-up examinations through the nationwide follow-up network.

Demographic data, medical information, and the socio-emotional status of the parents are documented in the case report form. Serial cerebral ultrasound (cUS) is used to detect, confirm, and monitor brain damage (Table 1). At follow-up, a blinded developmental pediatrician, who is not informed if the infant has received music therapy or not, conducts the measurements.

### Statistical analysis

The primary objective of this study is feasibility including the estimation of drop-out rate and differential drop-out rate within treatment groups. Time points for the evaluation of drop-out rates will be at 38–42 weeks and at 2 years. Drop-out rates and their corresponding confidence intervals will be estimated using the Wilson method [66].The success of recruitment will also be measured by summarizing randomization rates and reasons of withdrawal compared to available patients listed in the screening log. We anticipate differential protocol adherence with respect to treatment group assigned.

To test the effect of the intervention, point estimates of mean differences for efficacy will be estimated with corresponding 95% confidence interval for continuous outcomes, and differences in proportions with 95% confidence interval for the two treatment groups will be calculated for categorical outcomes. Univariate analysis of covariance (ANCOVA) will be performed to examine MRI differences between the groups. The independent variables are treatment group and gender. Resulting estimates of mean differences and differences in proportions including variability will serve to perform the sample size calculation for the multi-center full-scale trial.

## Discussion

Our initial experience with recruiting and consenting the parents has revealed parental concerns regarding the fact that their infant could end up in the control group and would not receive the music therapy intervention. Indeed, for some, this was a motivating factor not to participate in the study. We also had to exclude some control infants from the study because the parents started to intuitively sing to their infants (or they previously done so on a regular basis). These potential confounders highlight the value that many parents place on music therapy and the role of singing in interactions with their babies and the bonding process as previously reported in the literature [48, 67].

Although approximately 120 preterm infants under 32 SSW are treated in the unit every year, recruiting time is much longer as expected since approximately 60% of the patients treated in our unit are from other countries and cultures and study participation is often limited because of language and ethnical barriers [68]. Culturally congruent study information available in other languages may reduce some of these barriers in future investigations, and a multi-center study is warranted to recruit enough participants for a full-scale trial in an appropriate time span. Also, infants with severe brain injury such as intraventricular hemorrhage (grades III–IV) or cystic white matter injury are not included into the study.

It is worthwhile to note that double-blinding was not possible given the intervention design. The goal is to evaluate responsive, interactive live music therapy rather than simply comparing a standardized-recorded auditory stimulus which could be relatively easy to conduct using double-blind headphones with/without music stimulus. Indeed, neurobehavioral development is thought to be accelerated by nurturing, social learning experiences, and human interaction as opposed to machine-based interactions [42, 69].

To our knowledge, this is the first randomized controlled clinical trial to systematically investigating the potential short- and long-term effects of CMT on brain development in preterm infants. This project lies at the interface of music therapy, neuroscience, and medical imaging. New insights into the potential role and impact of music on brain function and development may be elucidated by this study. If such a low-cost, low-risk intervention is demonstrated in a future multi-center trial to be effective in supporting brain development in preterm neonates, findings could have broad clinical implications for this vulnerable patient population.

## Additional files

Additional file 1: Appendix 1. (PDF 99 kb)

Additional file 2: Appendix 2. (PDF 61 kb)

Additional file 3: Appendix 3. (PDF 48 kb)

## Abbreviations

ABAS: Adaptive Behavior Assessment System
KABC: Kaufman Assessment Battery for Children
BRIEF-P: German speaking adaptation of the Behavior Rating Inventory of Executive Function - Preschool Version
BSID-III: Bayley Scales of Infant and Toddler Development III
CGA: Corrected gestational age
CMT: Creative music therapy
cUS: Cerebral ultrasound
DTI: Diffusion tensor imaging
MRI: Magnet resonance imaging
NICU: Neonatal intensive care unit
PWMI: Periventricular white matter injury
rsFMRI: Resting state functional magnetic resonance imaging
SDQ: Strengths and Difficulties Questionnaire
SES: Socio-emotional status of the parents
TBSS: Tract-based spatial statistics

## References

[1] March of Dimes, PMNCH, Save the Children, WHO (2012). Born too soon.

[2] Keller M, Saugstad O, Steenbrugge, Mader S, Thiele N. Caring for tomorrow: EFCNI White Paper on Maternal and Newborn Health and Aftercare Services. European Foundation for the Care of Newborn Infants. 2011;195.

[3] Boardman JP, Counsell SJ, Rueckert D, Kapellou O, Bhatia KK, Aljabar P (2006). Abnormal deep grey matter development following preterm birth detected using deformation-based morphometry. NeuroImage.

[4] Inder TE, Hüppi PS (1999). Periventricular white matter injury in the premature infants id followed by reduced cerebral cortical gray matter volume at term. Ann Neurol.

[5] Dubois J, Benders M, Borradori-Tolsa C, Cachia A, Lazeyras F, Ha-Vinh Leuchter R (2008). Primary cortical folding in the human newborn: an early marker of later functional development. Brain.

[6] Volpe JJ (2009). Brain injury in premature infants: a complex amalgam of destructive and developmental disturbances. Lancet Neurol.

[7] Woodward LJ, Anderson PJ, Austin NC, Howard K, Inder TE (2006). Neonatal MRI to predict neurodevelopmental outcomes in preterm infants. N Engl J Med.

[8] Wehrle FM, Kaufmann L, Benz LD, Huber R, O’Gorman RL, Latal B (2016). Very preterm adolescents show impaired performance with increasing demands in executive function tasks. Early Hum Dev.

[9] Smith GC, Gutovich J, Smyser C, Pineda R, Newnham C, Tjoeng TH (2011). Neonatal intensive care unit stress is associated with brain development in preterm infants. Ann Neurol.

[10] Anand KJS, Scalzo FM (2000). Can adverse neonatal experiences alter brain development and subsequent behavior?. Biol Neonate.

[11] Kato T, Okumura A, Hayakawa F, Tsuji T, Natsume J, Watanabe K (2011). Evaluation of brain maturation in pre-term infants using conventional and amplitude-integrated electroencephalograms. Clin Neurophysiol.

[12] Lahav A, Skoe E. An acoustic gap between the NICU and womb: a potential risk for compromised neuroplasticity of the auditory system in preterm infants. Front Neurosci. 2014;8:381. 10.3389/fnins.2014.00381 PMC4256984 25538543

[13] McMahon E, Wintermark P, Lahav A (2012). Auditory brain development in premature infants: the importance of early experience. Ann N Y Acad Sci.

[14] Neville H, Bavelier D. Human brain plasticity: evidence from sensory deprivation and altered language experience. Prog Brain Res. 2002;138:177–88. 10.1016/S0079-6123(02)38078-6 12432770

[15] Trevarthen C (2008). The musical art of infant conversation: narrating in the time of sympathetic experience, without rational interpretation, before words. Music Sci.

[16] Schore AN, Wilkinson JC, H (2003). Minds in the making: attachment, the self-organising brain, and developmentally oriented psychoanalytic psychotherapy. Revolutionary connections: psychotherapy and neuroscience.

[17] Als H, Duffy FH, McAnulty G, Butler SC, Lightbody L, Kosta S (2012). NIDCAP improves brain function and structure in preterm infants with severe intrauterine growth restriction. J Perinatol.

[18] Dahmen JC, King AJ (2007). Learning to hear: plasticity of auditory cortical processing. Curr Opin Neurobiol.

[19] de Villers-Sidani E, Simpson KL, Lu YF, Lin RC, Merzenich MM (2008). Manipulating critical period closure across different sectors of the primary auditory cortex. Nat Neurosci.

[20] Chang EF, Merzenich MM (2003). Environmental noise retards auditory cortical development. Science (80-).

[21] Yan J (2003). Canadian Association of Neuroscience Review: development and plasticity of the auditory cortex. Can J Neurol Sci.

[22] Angelucci F, Ricci E, Padua L, Sabino A, Tonali PA (2007). Music exposure differentially alters the levels of brain-derived neurotrophic factor and nerve growth factor in the mouse hypothalamus. Neurosci Lett.

[23] Xu J, Yu L, Cai R, Zhang J, Sun X (2009). Early auditory enrichment with music enhances auditory discrimination learning and alters NR2B protein expression in rat auditory cortex. Behav Brain Res.

[24] Shoemark H, Hanson-Abromeit D, Stewart L. Constructing optimal experience for the hospitalized newborn through neuro-based music therapy. Front Hum Neurosci. 2015;9:487. 10.3389/fnhum.2015.00487 PMC4558927 26388762

[25] Abbott A (2002). Music, maestro, please!. Nature.

[26] Rickard NS, Toukhsati SR, Field SE (2005). The effect of music on cognitive performance: insight from neurobiological and animal studies. Behav Cogn Neurosci Rev.

[27] Sacks O (2007). Tales of music and the brain.

[28] Koelsch S (2014). Brain correlates of music-evoked emotions. Nat Publ Gr.

[29] Koelsch S (2014). Brain correlates of music-evoked emotions. Nat Rev Neurosci.

[30] Fachner J, Gold C, Erkkila J (2013). Music therapy modulates fronto-temporal activity in rest-EEG in depressed clients. Brain Topogr.

[31] Lin S-T, Yang P, Lai C-Y, Su Y-Y, Yeh Y-C, Huang M-F (2011). Mental health implications of music: insight from neuroscientific and clinical studies. Harv Rev Psychiatry.

[32] Sakatani K, Chen S, Lichty W, Zuo H, Wang YP (1999). Cerebral blood oxygenation changes induced by auditory stimulation in newborn infants measured by near infrared spectroscopy. Early Hum Dev.

[33] Perani D, Saccuman MC, Scifo P, Spada D, Andreolli G, Rovelli R (2010). Functional specializations for music processing in the human newborn brain. Proc Natl Acad Sci USA.

[34] Lecanuet JP, Schaal B (1996). Fetal sensory competencies. Eur J Obs Gynecol Reprod Biol.

[35] Huotilainen M, Näätänen R. Auditory perception and early brain development. Encyclopedia on Early Childhood Development. 2010:1–5.

[36] Partanen E, Kujala T, Naatanen R, Liitola A, Sambeth A, Huotilainen M (2013). Learning-induced neural plasticity of speech processing before birth. Proc Natl Acad Sci.

[37] Koelsch S (2009). A neuroscientific perspective on music therapy. Ann N Y Acad Sci.

[38] Koelsch S (2010). Towards a neural basis of music-evoked emotions. Trends Cogn Sci.

[39] Nordoff P, Robbins C (1977). Creative music therapy: individualized treatment for the handicapped child.

[40] Haslbeck F (2004). Music therapy with preterm infants—theoretical approach and first practical experience. Music Therapy Today.

[41] Haslbeck FB (2013). Creative music therapy with premature infants: an analysis of video footage. Nord J Music Ther.

[42] Loewy J (2015). NICU music therapy: song of kin as critical lullaby in research and practice. Ann N Y Acad Sci.

[43] Hanson-Abromeit D, Shoemark H, Loewy J, Hanson-Abromeit D, Colwell C (2008). Music therapy with pediatric units: newborn intensive care unit (NICU). Medical music therapy for pediatrics in hospital settings. Using music to support medical interventions.

[44] Fischer G, Riedesser P (2009). Lehrbuch der Psychotraumatologie [textbook of psychotraumatology].

[45] Bieleninik L, Ghetti C, Gold C (2016). Music therapy for preterm infants and their parents: a meta-analysis. Pediatrics.

[46] Haslbeck FB (2012). Music therapy for premature infants and their parents: an integrative review. Nord J Music Ther.

[47] Standley J (2012). Music therapy research in the NICU: an updated meta-analysis. Neonatal Netw.

[48] Haslbeck FB (2014). The interactive potential of creative music therapy with premature infants and their parents: a qualitative analysis. Nord J Music Ther.

[49] Patel AD (2008). Music, language and the brain.

[50] Shih WJ, Ohman-Strickland PA, Lin Y (2004). Analysis of pilot and early phase studies with small sample sizes. Stat Med.

[51] Browne RH (1995). On the use of a pilot sample for sample size determination. Stat Med.

[52] Haslbeck F, Costes T (2011). Advanced training in music therapy with premature infants—impressions from the United States and a starting point for Europe. Br J Music Ther.

[53] Lepomäki V, Leppänen M, Matomäki J, Lapinleimu H, Lehtonen L, Haataja L (2013). Preterm infants’ early growth and brain white matter maturation at term age. Pediatr Radiol.

[54] O’Gorman RL, Bucher HU, Held U, Koller BM, Hüppi PS, Hagmann CF (2015). Tract-based spatial statistics to assess the neuroprotective effect of early erythropoietin on white matter development in preterm infants. Brain.

[55] Gui L, Lisowski R, Faundez T, Hüppi PS, Lazeyras F, Kocher M (2012). Morphology-driven automatic segmentation of MR images of the neonatal brain. Med Image Anal.

[56] Johnson S, Wolke D, Marlow N (2008). Developmental assessment of preterm infants at 2 years: validity of parent reports. Dev Med Child Neurol.

[57] Largo RH, Fischer JE, Caflisch JA, Jenni OG (2007). Zürcher Neuromotorik.

[58] Palisano R, Rosenbaum P, Walter S, Russell D, Wood E, Galuppi B (1997). Development and reliability of a system to classify gross motor function in children with cerebral palsy. Dev Med Child Neurol.

[59] Mikrostrabismus LJ (1982). Die Bedeutung der Mikrotropie für Amblyopie, für die Pathogenese des grossen Schielwinkels und für die Heredität des Strabismus.

[60] Harrison P, Oakland T (2003). Adaptive behavior assessment system (ABAS II).

[61] Marlow N, Hennessy EM, Bracewell MA, Wolke D (2007). Motor and executive function at 6 years of age after extremely preterm birth. Pediatrics.

[62] Melchers P (2009). Preuss. Kaufman Assessment Battery for Children (German version).

[63] GOODMAN R (2017). Psychometric properties of the strengths and difficulties questionnaire. J Am Acad Child Adolesc Psychiatry.

[64] Adams M, Grunt S, Weber P (2014). A CM. Follow-up assessment of high-risk newborns in Switzerland. Paediatrica.

[65] Daseking M, Petermann F (2013). Deutschsprachige Adaptation des Behavior Rating Inventory of Executive function–Preschool Version (BRIEFP) von GA Gioia, KA Espy & PK Isquith. itle.

[66] Wilson EB (1927). Probable inference, the law of succession, and statistical inference. J Am Stat Assoc.

[67] Shoemark H, Arnup S (2014). A survey of how mothers think about and use voice with their hospitalized newborn infant. J Neonatal Nurs.

[68] George S, Duran N, Norris K (2014). A systematic review of barriers and facilitators to minority research participation among African Americans, Latinos, Asian Americans, and Pacific Islanders. Am J Public Health.

[69] Shoemark H, Hanson-Abromeit D, Wheeler B (2015). Music therapy in the neonatal intensive care unit. Music therapy handbook.

