# Supplementary material for: Creative music therapy to promote brain structure, function, and neurobehavioral outcomes in preterm infants: a randomized controlled pilot trial protocol
Source: Pilot Feasibility Stud. 2017 Sep 26;3:36. doi: 10.1186/s40814-017-0180-5 (PMC5613472; doi:10.1186/s40814-017-0180-5)
Supplement: Supplementary file 1 — Appendix 1. (PDF 99 kb) [file 40814_2017_180_MOESM1_ESM.pdf]

Informationsblatt für die Eltern

Studientitel: Schöpferische Musiktherapie für Frühgeborene:
Mögliche positive Auswirkungen auf Gehirnstruktur, -funktion und die weitere neurologische Entwicklungen der Kinder (Musikstudie FG)

Liebe Eltern,

1. Auswahl der Studienteilnehmer

Sie wurden für die Studie angefragt, weil Ihr Kind als Frühgeborenes auf unserer neonatologischen Station versorgt wird und vor 32 vollendeten Schwangerschaftswochen geboren wurde.

2. Ziel der Erhebung

Bei dem vorliegenden Forschungsvorhaben geht es um Musiktherapie für Frühgeborene auf der versorgenden Intensivstation. Musiktherapie ist im Frühgeborenenbereich eine neue unterstützende Therapieform, über die bislang erst wenige wissenschaftliche Erkenntnisse vorliegen. Ziel dieser Studie ist es, mögliche positive Auswirkungen auf die (Gehirn-) Entwicklung zu untersuchen.

3. Allgemeine Informationen zur Erhebung

Diese Studie wird nach geltenden schweizerischen Gesetzen und nach international anerkannten Grundsätzen durchgeführt. Sie wurde von der zuständigen, unabhängigen Ethikkommission des Kantons Zürich genehmigt. Falls Sie sich entscheiden, mit Ihrem frühgeborenen Kind an der Musik Studie teilzunehmen, wird Ihr Kind per Zufall in die Musikgruppe oder in die Kontrollgruppe eingeteilt werden. Die Kontrollgruppe erhält die Standardversorgung, die Musikgruppe zusätzlich Musiktherapie.

4. Freiwilligkeit der Teilnahme

Ihre Teilnahme an dieser Studie ist freiwillig. Wenn Sie auf die Teilnahme an dieser Studie verzichten, haben Sie keine Nachteile für die weitere medizinische Betreuung ihres Kindes zu erwarten. Das gleiche gilt, wenn Sie Ihre dazu gegebene Einwilligung zu einem späteren Zeitpunkt widerrufen. Diese Möglichkeit haben Sie jederzeit. Einen allfälligen Widerruf Ihrer Einwilligung bzw. den Rücktritt von der Studie müssen Sie nicht begründen. Im Falle eines Widerrufes werden die bis zu diesem Zeitpunkt erhobenen Daten weiter verwendet.

5. Studienablauf

Falls Sie sich entscheiden, mit Ihrem frühgeborenen Kind an der Musikstudie teilzunehmen, werden folgende Schritte unternommen:

- Ihr Kind wird per Zufall in die Kontrollgruppe oder in die Musikgruppe eingeteilt.
- Es werden Informationen zu Ihrem Kind (z.B. Geburtsdatum, Gewicht, Diagnose, Krankenhausverweildauer, routinemässige EEG- und Ultraschall-Untersuchungen) elektronisch erfasst.
- Wir werden im Alter von zirka 32 und 40 Schwangerschaftswochen eine studienbedingte Magnetresonanzuntersuchung (MR) bei Ihrem Kind durchführen, um die Entwicklung des Gehirnes beurteilen zu können. Die MR-Untersuchungen werden im natürlichen Schlaf durchgeführt, d.h. nachdem Ihr Kind zu trinken erhalten hat. Während der ganzen MR Untersuchung wird Ihr Kind beobachtet und einen Hörschutz erhalten. Wenn Ihr Kind vorzeitig erwachen sollte, erhält es einen Nuggi mit Zuckersirup, aber keine Medikamente oder Narkose.
- An den gleichen Tagen wie die MR-Untersuchungen mit 32 und 40 Wochen möchten wir während einer Stunde ein EEG ableiten, um präzise Information über die

Hirnentwicklung Ihres Kindes zu erhalten. Diese Untersuchung ist rein studienbedingt.
- Im Alter von korrigierten 9-12 Monaten, sowie mit 24 Monaten und im Alter von 5 Jahren werden Sie zu einer entwicklungsneurologischen Kontrolle aufgeboten. Diese Untersuchungen sind Routineuntersuchungen für alle frühgeborenen Kinder in der Schweiz und werden zur Qualitätskontrolle gebraucht. Die Ergebnisse möchten wir zusätzlich für diese Studie verwenden.
- Wird Ihr Kind in die Musikgruppe eingeteilt, erhält es während der Zeit auf der neonatologischen Intensivstation dreimal wöchentlich Musiktherapie. für ca. 20 Minuten. Es werden einfache, leise Melodien auf dem Atemrhythmus Ihres Kindes gesungen, um die Atmung zu unterstützen, Ihr Kind zu beruhigen und gleichzeitig sanft zu fördern. Zusätzlich sollen Sie als Eltern unterstützt werden, Kontakt zu Ihrem Kind auch über Ihre eigene Stimme aufzubauen. Wir möchten Sie bitten, Ihr Einverständnis zu geben, Ihr Kind während ein bis zwei Musiktherapiesitzungen mit der Videokamera filmen zu dürfen. Das Videomaterial wird in anonymisierter Form abgespeichert.
- Wenn Ihr Kind in die Kontrollgruppe eingeteilt wird, erhält es die übliche Behandlung und Zuwendung und es wird einen Hörschutz erhalten, wenn daneben ein Kind Musiktherapie erhält.

6. Pflichten der PrüfärztInnen und Vertraulichkeit der Daten
Die Studie ist von der zuständigen Ethik-Kommission begutachtet und bewilligt worden. Die Daten Ihres Kindes werden anonymisiert ausgewertet. Während der ganzen Studie wird die Vertraulichkeit strikt gewahrt. Der Name Ihres Kindes wird in keiner Weise in Publikationen, die aus der Studie hervorgehen, veröffentlicht. Alle am Projekt beteiligten Personen unterliegen einer strikten Schweigepflicht.

7. Nutzen für die Teilnehmenden
Ihr Kind wird während seines Aufenthaltes im UniversitätsSpital Musiktherapie erhalten. Spezialisierte Fachärzte werden die MRI und EEG Befunde beurteilen. Die betreuenden Ärzte werden Sie darüber informieren. Dank Ihrer Studienteilnahme können die Ergebnisse auch anderen Frühgeborenen und deren Betreuung zu Gute kommen.
Wenn eine MR-Untersuchung nicht ohnehin klinisch indiziert ist, erhält Ihr Kind eine solche kostenlos. Wenn Sie dies wünschen werden Sie die betreuenden Ärzte über die Ergebnisse informieren. Wenn Sie an der Studie teilnehmen, hat Ihr Kind eine 50%-Chance, eine kostenlose Musiktherapie zu erhalten.

8. Risiken und Unannehmlichkeiten
Da ausschliesslich nicht-invasive Verfahren verwendet und keine Medikamente verabreicht werden, beinhaltet diese Studie nach unserem besten Wissen für Ihr Kind kein Risiko.

9. Neue Erkenntnisse
Die Erkenntnisse dieser Musikstudie werden in öffentlich zugänglichen medizinischen Fachzeitschriften veröffentlicht. Wenn Sie dies wünschen, werden wir Sie darüber auch persönlich informieren.
Werden bei den MR-Untersuchungen Ihres Kindes zufällig neue Befunde erhoben, die zur Verhinderung, Feststellung und Behandlung von Krankheiten beitragen könnten, haben Sie die Wahl: a) Sie möchten über diese Befunde direkt informiert werden, b) Sie möchten nicht informiert werden, oder c) Sie überlassen die Entscheidung Ihrem behandelnden Arzt/Ärztin Sie können Ihren Wunsch mit der schriftlichen Einverständniserklärung angeben, aber auch später noch ändern.

10. Kosten / Entschädigung für die Studienteilnehmenden
Die in dieser Patienten-Information erwähnten studienspezifischen Untersuchungen sind kostenlos. Weder Ihnen noch ihrer Krankenkasse entstehen im Zusammenhang mit Ihrer Teilnahme zusätzliche Kosten. Für die Teilnahme an dieser Erhebung erhalten Sie keine

finanzielle Entschädigung. Wir vergüten Ihnen jedoch auf Wunsch die Reisespesen für die entwicklungsneurologischen Untersuchungen.

11. Unfreiwilliger Studienabbruch

Sowohl die Musiktherapie als auch die MR-Untersuchung werden unterbrochen oder abgebrochen wenn ein Kind dabei unruhig wird oder sonstige Anzeichen bestehen, dass es diese nicht erträgt.

12. Deckung von Schäden

Treten während oder nach dem klinischen Versuch gesundheitliche Störungen oder irgendwelche Schäden auf, so wenden Sie sich bitte an den verantwortlichen Studienarzt (Prof. Dr. H.U. Bucher). Er wird für Sie die notwendigen Schritte einleiten.
Die Betriebshaftpflichtversicherung des UniversitätsSpitals ersetzt Ihnen Schäden, die Sie gegebenenfalls im Rahmen des klinischen Versuchs erleiden.

Kontaktpersonen

Bei Unklarheiten, Notfällen, unerwarteten oder unerwünschten Ereignissen, die während der Studie oder nach deren Abschluss auftreten, können Sie sich jederzeit an die untenstehenden Kontaktpersonen wenden:

Prof. em. Dr. med. H. U. Bucher	Telefon 044 255 53 40	Email: buh@usz.ch
Dr. F. Haslbeck		Email: friederike.haslbeck@usz.ch
Prof. Dr. med. D. Bassler	Telefon 044 255 53 40	Email: dirk.bassler@usz.ch
PD Dr. med. C. Hagmann	Telefon 044 255 53 45	Email: cornelia.hagmann@usz.ch

Gemeinsame Adresse: Klinik für Neonatologie, UniversitätsSpital Zürich, 8091 Zürich

Telefonnummer mit 24h Erreichbarkeit: 044 255 53 51
