# Supplementary material for: Creative music therapy to promote brain structure, function, and neurobehavioral outcomes in preterm infants: a randomized controlled pilot trial protocol
Source: Pilot Feasibility Stud. 2017 Sep 26;3:36. doi: 10.1186/s40814-017-0180-5 (PMC5613472; doi:10.1186/s40814-017-0180-5)
Supplement: Supplementary file 2 — Appendix 2. (PDF 61 kb) [file 40814_2017_180_MOESM2_ESM.pdf]

Schriftliche Einverständniserklärung der Eltern oder der sorgeberechtigten Person zur Teilnahme ihrer Kinder an einer klinischen Studie

- Bitte lesen Sie dieses Formular sorgfältig durch.
- Bitte fragen Sie, wenn Sie etwas nicht verstehen oder wissen möchten.

**Titel der Studie:**	Schöpferische Musiktherapie für Frühgeborene: Mögliche positive Auswirkungen auf Gehirnstruktur, -funktion und die weitere neurologische Entwicklung der Kinder
**Sponsor**	Prof. em. H.U. Bucher<br>Klinik für Neonatologie<br>UniversitätsSpital Zürich<br>8091 Zürich
**Ort der Studie:**	Klinik für Neonatologie<br>Frauenklinikstrasse 10<br>8091 Zürich
**Prüfer:**<br>Name und Vorname:	Prof. em. H.U. Bucher, Prof. D. Bassler, PD Dr. C. Hagmann, PD Dr. G.C. Natalucci und Dr. Friederike Haslbeck
**Kind:**	Name und Vorname:<br>Geburtsdatum:

- Ich wurde vom unterzeichnenden Prüfer mündlich und schriftlich über die Ziele, den Ablauf der Erhebung, über die zu erwartenden Wirkungen, über mögliche Vor- und Nachteile sowie über eventuelle Risiken informiert.
- Ich habe die zur oben genannten Studie abgegebene schriftliche Elterninformation (Version 1 vom 19.11.2014) gelesen und verstanden. Meine Fragen im Zusammenhang mit der Teilnahme an dieser Erhebung sind mir zufriedenstellend beantwortet worden. Ich kann die schriftliche Elterninformation behalten und erhalte eine Kopie meiner schriftlichen Einverständniserklärung.
- Ich hatte genügend Zeit, um meine Entscheidung zu treffen.
- Ich weiss, dass die persönlichen Daten nur in anonymisierter Form an aussenstehende Institutionen zu Forschungszwecken weitergegeben werden. Ich bin einverstanden, dass die zuständigen Fachleute des Studienauftraggebers, der Behörden und der Kantonalen Ethikkommission zu Prüf- und Kontrollzwecken in meine Originaldaten Einsicht nehmen dürfen, jedoch unter strikter Einhaltung der Vertraulichkeit.
- Ich gebe die Einwilligung zur Erhebung freiwillig. Ich kann jederzeit und ohne Angabe von Gründen meine Zustimmung zur Teilnahme meines Kindes widerrufen, ohne dass meinem Kind deswegen Nachteile bei der weiteren medizinischen Betreuung entstehen.
- Ich bin darüber informiert, dass eine Versicherung Schäden deckt, falls solche im Rahmen der Studie auftreten.
- Bei Zufallsbefunden bei den MR-Untersuchungen mit 32 und 40 Schwangerschaftswochen möchte ich a) ☐ direkt informiert werden b) ☐ nicht informiert werden c) ☐ die Entscheidung dem behandelnden Arzt überlassen.

Ort, Datum	Unterschrift der Eltern/ der sorgeberechtigten Person

Bestätigung des Prüfers: Hiermit bestätige ich, dass ich den Eltern/ der sorgeberechtigten Person Wesen, Bedeutung und Tragweite der Erhebung erläutert habe. Ich versichere, alle im Zusammenhang mit dieser Studie stehenden Verpflichtungen zu erfüllen. Sollte ich zu irgendeinem Zeitpunkt während der Durchführung der Studie von Aspekten erfahren, welche die Bereitschaft der Eltern/ der sorgeberechtigten Person zur Teilnahme an der Studie beeinflussen könnten, werde ich sie umgehend darüber informieren.

Ort, Datum	Unterschrift der Prüfers
