# Supplementary material for: Creative music therapy to promote brain structure, function, and neurobehavioral outcomes in preterm infants: a randomized controlled pilot trial protocol
Source: Pilot Feasibility Stud. 2017 Sep 26;3:36. doi: 10.1186/s40814-017-0180-5 (PMC5613472; doi:10.1186/s40814-017-0180-5)
Supplement: Supplementary file 3 — Appendix 3. (PDF 48 kb) [file 40814_2017_180_MOESM3_ESM.pdf]

Protocol

**Session, number:**	**Date:**		**Time:**
**Video:**			
**Position:**			
**Incubator:**	**Warmer:**	**Handling:**	**Cradling:**
**Parents` integration:**			

Before MT	Infant	Parents/ Environment
**Behavioral State**		
**Physiological data**	**Heart rate:** **Oxygen saturation:** **Respiration rate:**	
**Other comments**		

During MT	Infant	Parents / Env.
**Behavioral State**		
**Physiological data**	**Heart rate:** **Oxygen saturation:** **Respiration rate:**	
**Breathing**		
**Motoric**		
**Mimic/ gesture**		
**Sucking**		
**Vocalizing**		

After MT	Infant	Parent / Env.
**Behavioral State**		
**Physiological d.**	**Heart rate:** **Oxygen saturation:** **Respiration rate:**	
**Other comments**		

Parents` conversation protocol

**Date:**	**Duration:**
**Location:**	
**Circumstances:**	
**Content:**	

* Protocol sheet translated in English for publication` s purpose
